# On Metric Dimension in Some Hex Derived Networks †

**DOI:** 10.3390/s19010094

**Published:** 2018-12-28

**Authors:** Zehui Shao, Pu Wu, Enqiang Zhu, Lanxiang Chen

**Affiliations:** 1 Institute of Computing Science and Technology, Guangzhou University, Guangzhou 510006, China; zshao@gzhu.edu.cn (Z.S.); puwu1997@126.com (P.W.); zhuenqiang@pku.edu.cn (E.Z.); 2 College of Mathematics and Informatics, Fujian Normal University, Fujian Provincial Key Laboratory of Network Security and Cryptology, Fujian Network & Information Security Industry Technology Development Base, Fuzhou 350117, China

**Keywords:** robot navigation, sensor network, metric dimension, metric basis

## Abstract

The concept of a metric dimension was proposed to model robot navigation where the places of navigating agents can change among nodes. The metric dimension md(G) of a graph *G* is the smallest number *k* for which *G* contains a vertex set *W*, such that |W|=k and every pair of vertices of *G* possess different distances to at least one vertex in *W*. In this paper, we demonstrate that md(HDN1(n))=4 for n≥2. This indicates that in these types of hex derived sensor networks, the least number of nodes needed for locating any other node is four.

## 1. Introduction

A task in robot navigation is to obtain the position immediately, whenever we want to know it. Suppose that a robot navigating in a sensor network is able to automatically obtain the distances to a collection of landmarks, then we can find a subset of nodes in the network such that the robot’s position in the network is uniquely identified. In order to achieve this, the concept of “landmarks in a graph” was developed [1], and later was extended to the “metric dimension”, in which one considers networks in the graph-structure framework.

Let $R^{k}$ be a *k*-dimensional Euclidean space and Z be the integer set. Assume $Z^{k}=\left\{\left(x_{1},x_{2},\cdots ,x_{k}\right)\in R^{k}|x_{i}\in Z,1\leq i\leq k\right\}$. Every graph we consider is simple and connected and contains neither multiple edges nor loops. For two vertices $v_{1},v_{2}\in V\left(G\right)$ of a graph G=(V(G),E(G)), we denote by $d_{G}\left(v_{1},v_{2}\right)$ (or simply by $d(v_{1},v_{2})$) the *distance* between $v_{1}$ and $v_{2}$, i.e., the number of edges in the shortest path from $v_{1}$ to $v_{2}$. For a positive integer t≥1, we call *u* a *t*-neighbor of *v* if d(u,v)=t. We call the set $N_{t}\left(v\right)=\left\{s\in V\left(G\right)|d\left(v,s\right)=t\right\}$ the *t-neighbourhood of v*, and let $N_{t}^{-}\left(v\right)=\left\{s\in V\left(G\right)|d\left(v,s\right)\leq t\right\}$ and $N_{t}^{+}\left(v\right)=\left\{s\in V\left(G\right)|d\left(v,s\right)\geq t\right\}$. In particular, $N_{1}\left(v\right)$ is called the *open neighborhood* of *v* and simply denoted by N(v), and N[v]=N(v)∪{v} is the *closed neighborhood* of *v*. The *degree* of a vertex *v* is the cardinality of N(v) and denoted by deg(v).

Given a positive integer *k* and an ordered set $S=\left\{s_{1},s_{2},\cdots ,s_{k}\right\}\subseteq V\left(G\right)$, for a vertex t∈V(G), we regard the *k*-vector $\xi \left(t|S\right)=(d\left(t,s_{1}\right),d\left(t,s_{2}\right),\cdots ,d\left(t,s_{k}\right))$ as the *metric representation of v with respect to S*. If any two distinct vertices of *G* do not have the identical representation with respect to *S*, then we call *S* a *resolving set* (RS) of *G*. The *metric basis* of *G* is the RS of *G* with the smallest cardinality. A metric basis of cardinality *k* is also called a *k-metric basis*. The metric dimension of *G*, denoted by md(G), is defined as the cardinality of a metric basis.

For convenience, we summarize the symbols we use in Table 1.

Due to their important applications and theoretical studies, various versions of metric generators have been proposed, which contribute deep insights into the mathematical properties of the metric dimension involving distances in graphs. Many authors have introduced different variations of metric generators—such as independent resolving sets [2], local metric sets [3], resolving dominating sets [4], strong resolving sets [5], *k*-metric generators [6], and a mixed metric dimension [7]—and their properties have been studied.

The subject of determining md(G) of a graph *G* was initially studied by Harary, et al. [8], and Slater [9] independently proved that determining md(G) of a graph *G* is an NP-complete problem [10]. The metric dimension has been extensively studied not merely for the computational intractability, but also for its applications in many fields, such as robot navigation [1], telecommunication, chemistry [2,11], and combinatorial optimization [4,12,13,14,15,16,17], among many others.

Honeycomb networks are a variant of meshes and tori that play an essential role in the areas of image processing, cellular phone base stations, computer graphics, and mathematical chemistry [18,19,20], because they have more attractive structural properties with respect to their diameter, degree, the total number of edges, the bisection width, and cost. Stojmenovic [20] and Parhami [21] analyzed the topological descriptors of honeycomb networks and presented an united formulation for the honeycomb. In Reference [18], based on honeycomb and hexagonal meshes, Manuel et al. introduced two new hexagonal networks, which have more interesting properties and features over certain honeycomb networks and meshes. Manuel et al. [18] posed an interesting open question to determine whether the metric dimensions of these kinds of hex-derived networks (HDNs) are between three and five. Xu and Fan [22] gave a proof and showed that the metric dimensions of the hex-derived networks HDN1(n) and HDN2(n) are either three or four. However until now, the exact metric dimension of these networks is still unknown. In this paper, we solve this problem for HDN1 networks by showing that md(HDN1(n))=4 for n≥2.

The main contributions of this paper are listed as follows:

- We propose a vector coloring scheme to study properties of some networks with metric dimension three. By applying this approach we succeed to process hex-derived networks. Therefore, the proposed approach is a promising approach to determine if a network has metric dimension three.
- The hexagonal networks are popular mesh-derived parallel architectures, which are also a kind of sensor network and widely used in computer graphics and cellular phone base stations. Inspired by the important applications of hex-derived network, Manuel et al. started to study the metric dimension of hex-derived networks. They proposed an open problem to determine whether the metric dimension of a kind of hex-derived networks lies between three and five. Xu and Fan showed that it is less than five. In this paper, we apply our approach to completely solve this problem.

## 2. HDN1 Networks

**Figure 1:**
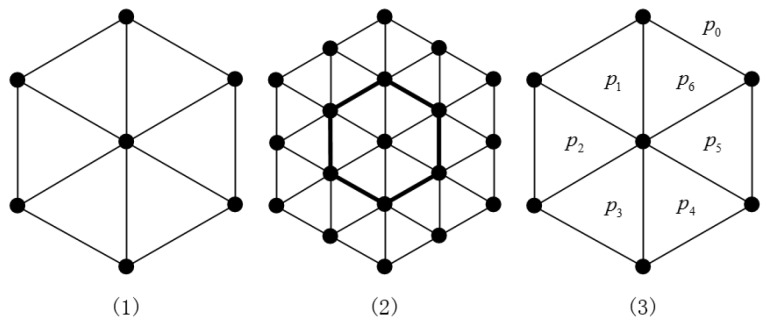
Schematics of *n*-dimensional hexagonal meshes, HX(*n*): (1) HX(2), (2) HX(3), and (3) all of the faces in HX(2).

In this section, we describe the definition of HDN1 networks. We follow the presentation in Reference [22]. The concept of a hexagonal mesh was introduced by Chen et al. [19]. Recall that a *planar graph* is a graph that can be drawn such that no edges cross each other. An *n*-dimensional hexagonal mesh for n≥2, denoted by HX(*n*), is a planar graph which consists of a collection of triangles as shown in Figure 1. The 2D hexagonal mesh HX(2) is made up of 6 triangles (see Figure 1(1)). The 3D hexagonal mesh HX(3) is constructed from HX(2) by including additional triangles around the boundary of HX(2) (see Figure 1(2)). Similarly, HX(*n*) is established by including additional triangles around the boundary of HX(n−1).

In a planar graph, there are many faces of *G*. If two faces *p* and *q* share at least one edge, they are said to be adjacent, or *p* is a neighbor of *q*. If a planar graph contains exactly one unbounded face, it is called the *outer face* of the graph. For example Figure 1(3) shows that HX(2) has seven faces $p_{0},p_{1},\ldots ,p_{6}$, for which $p_{1}$ is adjacent to $p_{0},p_{2}$ and $p_{6}$; and $p_{0}$ is an outer face. These definitions can be found in Reference [22].

**Figure 2:**
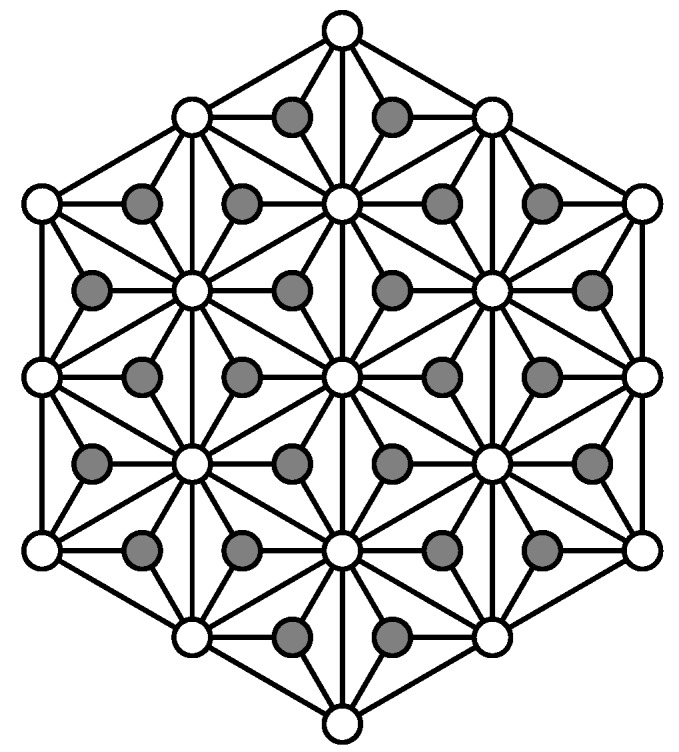
Hex-derived network, HDN1(3).

Given a graph HX(*n*), we use F(HX(n)) to denote the set of non-outer faces of HX(n). Now, for each p∈F, we add a new vertex $p^{*}$ which is located in the face *p* and connects $p^{*}$ with the three vertices of *p*. The resulting graph is HDN1(n). As an example, HDN1(3) can be found in Figure 2, where the gray vertices are the additional vertices based on HX(3).

Suppose that $p_{i}$ is a neighbor of *p* for each i=1,2,⋯,k and $p_{1}^{*},p_{2}^{*},\ldots ,p_{k}^{*}$ have a one-to-one mapping to $p_{1},p_{2},\ldots ,p_{k}$, respectively. If the vertices of *p* and $p_{1}^{*},p_{2}^{*},\ldots ,p_{k}^{*}$ are joined with $p^{*}$, then we obtain HDN2(n). It is clear that HDN2(n) contains HDN1(n) as a subgraph. For n≥2, we also view HDN1(n) and HDN2(n) collectively as HDN(n).

The central vertex of HDN(n) is denoted by $u_{0}=\left(0,0,0\right)$. For an integer *i*, we adopt the following notations:

$$\begin{array}{ccc} V_{i} & = & \{x\in V\left(G\right)|d\left(x,u_{0}\right)=i\},\\ D_{i} & = & \{x\in V(G)|deg(x)=i\},\\ U_{i} & = & V_{i}\cap D_{3}. \end{array}$$

**Figure 3:**
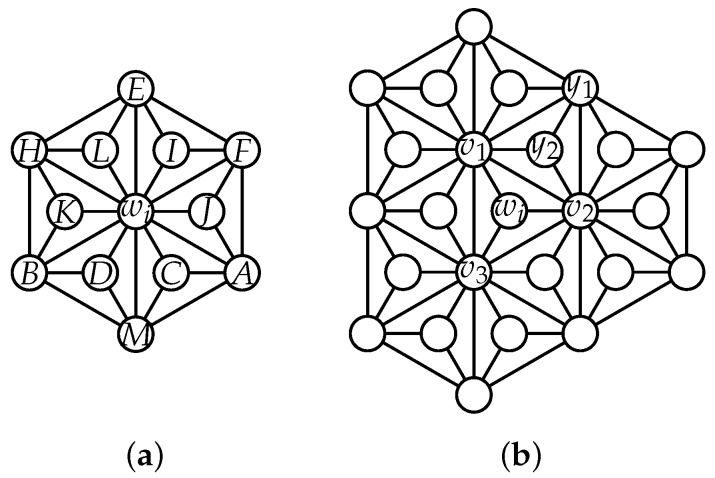
(**a**) HDN1(2) and (**b**) some vertices in HDN1(3).

In order to understand the idea clearly, we take the network described in Figure 3a as an example, which has the nodes $\left\{w_{i},A,B,C,D,E,F,H,I,J,K,L,M\right\}$. Assume we use S={A,M,B,H} as landmarks, then a robot, which knows the distances from each element in *S*, can obtain its own location in this network at any time. For instance, if the distance vector from (A,M,B,H) is (1,1,2,3), then it is located at the position *C* because the distance vectors from (A,M,B,H) are pairwise distinct.

## 3. Navigation in Certain Hex-Derived Sensor Networks

P. Manuel et al. [18] studied the navigation of certain hex-derived networks and proposed an open problem as follows.

**Open Problem**. Let *G* be HDN1 or HDN2, then is it true that 3≤md(G)≤5?

D. Xu [22] et al. have provide a proof to show that md(HDN(n)) is either 3 or 4, as shown in Theorems 1 and 2.

**Theorem** **1.** *[22] If n≥2, then we have md(HDN1(n))∈{3,4}.*

**Theorem** **2.** *[22] If n≥2, then we have md(HDN2(n))∈{3,4}.*

Note that the least number of nodes needed for locating any other node in such a network is unknown, we will solve this problem in this paper.

Let *G* be a graph and *W* be an ordered subset of V(G) with $W=\{w_{1},w_{2},\cdots ,w_{k}\}$. Assume $v_{0}\in V\left(G\right)$ and $\varphi \left(u,v_{0}\right)=\xi \left(u|W\right)-\xi \left(v_{0}|W\right)=\left(g_{1},g_{2},\cdots ,g_{k}\right)$ for any u∈V(G).

**Lemma** **1.** *For any e=xy∈E(G), we have $|g_{i}\left(x,v_{0}\right)-g_{i}\left(y,v_{0}\right)|\leq 1,i=1,2,\cdots ,k.$*

**Proof.**  Since $\varphi \left(x,v_{0}\right)=\xi \left(x|W\right)-\xi \left(v_{0}|W\right)$ and $\varphi \left(y,v_{0}\right)=\xi \left(y|W\right)-\xi \left(v_{0}|W\right)$, we have

$$|g_{i}\left(x,v_{0}\right)-g_{i}\left(y,v_{0}\right)\left|=\right|\left(d\left(x,w_{i}\right)-d\left(v_{0},w_{i}\right)\right)-\left(d\left(y,w_{i}\right)-d\left(v_{0},w_{i}\right)\right)|=|d\left(x,w_{i}\right)-d\left(y,w_{i}\right)|\leq 1.$$

 ☐

**Lemma** **2.** *Let t be a positive integer and v∈V(G). If $x,y\in N_{t}\left(v\right)$ and W is an RS of G, then ξ(x|W)≠ξ(y|W).*

**Definition** **1.** *Let md(G)=k and v∈V(G). A function $h:V\left(G\right)\to Z^{k-1}$ is said to be a (k−1)-vector coloring scheme on G with respect to v, if the following conditions are fulfilled:*

*(i)*  *For e=xy∈E(G) with $\alpha =\left(\alpha_{1},\alpha_{2},\cdots ,\alpha_{k-1}\right)=h\left(x\right)$ and $\beta =\left(\beta_{1},\beta_{2},\cdots ,\beta_{k-1}\right)=h\left(y\right)$, we have $|\alpha_{i}-\beta_{i}|\leq 1,i=1,2,\cdots ,k-1$.*
*(ii)*  *Let t>0. If $x,y\in N_{t}\left(v\right)$, we have h(x)≠h(y).*
*(iii)*  *h(v)=(0,0,⋯,0).*

**Lemma** **3.** *If there exists a subgraph $G^{\prime }$ of G and $v\in V(G^{\prime })$ such that $d_{G^{\prime }}\left(v,u\right)=d_{G}\left(v,u\right)$ for any $u\in V(G^{\prime })$, and there exists no (k−1)-vector coloring scheme on $G^{\prime }$ with respect to vertex v, then v is not in any k-metric basis of G.*

**Proof.**  Suppose that *W* is a *k*-metric basis of *G* with v∈W. Let $W=\{w_{1},w_{2},\cdots ,w_{k-1},v\}$ be an ordered set. For any u∈V(G), $\varphi \left(u,v\right)=\xi \left(u|W\right)-\xi \left(v|W\right)=\left(\varphi_{1},\varphi_{2},\cdots ,\varphi_{k}\right)$. Let $h\left(u\right)=\left(\varphi_{1},\varphi_{2},\cdots ,\varphi_{k-1}\right)$. By Lemmas 1 and 2, we have that *h* satisfies the three conditions of Definition 1, which yields a contradiction.  ☐

**Corollary** **1.** *Let W be a k-metric basis of G. If $G^{\prime }$ is a subgraph of G and $v\in V(G^{\prime })\cap W$ such that $d_{G^{\prime }}\left(v,u\right)=d_{G}\left(v,u\right)$ for any $u\in V(G^{\prime })$, then there must exist a (k−1)-vector coloring scheme on $G^{\prime }$ with respect to vertex v.*

Now, we will present the basic properties of hex-derived networks.

**Lemma** **4.** *Let n≥7. Suppose that md(HDN1(n))=3 and $W=\{w_{1},w_{2},w_{3}\}$ is an RS of HDN1(n), then we have $w_{i}\notin N_{n-1}^{-}\left(u_{0}\right)$, for any i∈{1,2,3}.*

**Proof.**  Suppose that there exists a $w_{i}\in N_{n-1}^{-}\left(u_{0}\right)$ for some *i*, then the following two cases are studied.  ☐

**Case** **1.** $w_{i}\in HX\left(n\right)$ (See Figure 3a).

Let $S=N[w_{i}]$ and $G^{\prime }=G\left[S\right]$. By Corollary 1, there exists a 2-vector coloring scheme *h* on $G^{\prime }$ with respect to vertex $w_{i}$. Let $h=(h_{1},h_{2})$. For any $u\in N_{1}\left(w_{i}\right)$, by Definition 1, we have $|h_{1}\left(u\right)|\leq 1$ and $|h_{2}\left(u\right)|\leq 1$. That is to say, $h_{1}\left(u\right),h_{2}\left(u\right)\in \left\{0,\pm 1\right\}$. Consequently, h(u) must be one of nine possible vectors for any $u\in N_{1}\left(w_{i}\right)$. Since $|N_{1}\left(w_{i}\right)|=12$, then there must exist two vertices $u_{1},u_{2}\in N_{1}\left(w_{i}\right)$ with $h\left(u_{1}\right)=h\left(u_{2}\right)$, which is a contradiction with the definition of *k*-vector coloring scheme (Definition 1).

**Case** **2.** $w_{i}\in D_{3}\cap HDN1\left(n\right))$ (See Figure 3b).

Let $S^{\prime }=N_{2}\left[w_{i}\right]$ and $G^{\prime }=G\left[S^{\prime }\right]$. Let $N_{1}\left(w_{i}\right)=\left\{v_{1},v_{2},v_{3}\right\}$. For any $u\in N_{1}^{-}\left(v_{1}\right)-\left\{w_{i}\right\}$, similarly, it can be proved that h(u) must be one of nine possible vectors. We have Claim 1 as follows.

**Claim** **1.** *There exists no three vertices $u_{1},u_{2},u_{3}\in N_{1}^{-}\left(v_{1}\right)-\left\{w_{i}\right\}$ with $h\left(u_{1}\right)=h\left(u_{2}\right)=h\left(u_{3}\right)$.*

**Proof** **of** **Claim** **1:** Suppose the statement does not hold. Since $N_{1}^{-}\left(v_{1}\right)-\left\{w_{i}\right\}\subset N_{1}\left(w_{i}\right)\cup N_{2}\left(w_{i}\right)$, then $u_{1},u_{2},u_{3}\in N_{1}\left(w_{i}\right)\cup N_{2}\left(w_{i}\right)$. Consequently, there must exist two vertices, say $u_{1},u_{2}\in N_{1}\left(w_{i}\right)$ or $u_{1},u_{2}\in N_{2}\left(w_{i}\right)$, which is a contradiction with Definition 1.

Since $|N_{1}^{-}\left(v_{1}\right)-\left\{w_{i}\right\}|=12$ and there are nine possibilities for *h*, then there must exist three pairs of vertices whose *h* values are equivalent. Note that $\left(N_{1}^{-}\left(v_{1}\right)-\left\{w_{i}\right\}\right)\cap N_{1}\left(w_{i}\right)=\left\{v_{1},v_{2},v_{3}\right\}$. From Definition 1, we know that the *h* values in $N_{t}\left(w_{i}\right)$ are distinct for positive integer t=1,2. Therefore it can be assumed that the three pairs of vertices are $\left(v_{1},x_{1}\right)$, $\left(v_{2},x_{2}\right)$, and $\left(v_{3},x_{3}\right)$ with $v_{1},x_{1},v_{2},x_{2},v_{3},x_{3}\in N_{1}^{-}\left(v_{1}\right)-\left\{w_{i}\right\}$ and that $h\left(v_{i}\right)=h\left(x_{i}\right),$ for i=1,2,3.

Similarly, there are three pairs of vertices $\left(v_{1},x_{1}^{\prime }\right)$, $\left(v_{2},x_{2}^{\prime }\right)$ and $\left(v_{3},x_{3}^{\prime }\right)$ with $v_{1},x_{1}^{\prime },v_{2},x_{2}^{\prime },v_{3},x_{3}^{\prime }\in N_{1}^{-}\left(v_{2}\right)-\left\{w_{i}\right\}$ and with $h\left(v_{i}\right)=h\left(x_{i}^{\prime }\right),$ for i=1,2,3.

From $h\left(x_{i}\right)=h\left(v_{i}\right)=h\left(x_{i}^{\prime }\right),$ for i=1,2,3, we have $x_{i}=x_{i}^{\prime }$. However, $|\left(N_{1}^{-}\left(v_{1}\right)-\left\{w_{i}\right\}\right)\cap \left(N_{1}^{-}\left(v_{2}\right)-\left\{w_{i}\right\}\right)\cap N_{2}\left(w_{i}\right)\left|=\right|\left\{y_{1},y_{2}\right\}|=2$, which yields a contradiction.  ☐

In the following, we discuss the vertices in $N_{n}\left(u_{0}\right)$ of HDN1(n): Let

(1) $$A=\{v\in N_{n}\left(u_{0}\right)\cap D_{3}:|N\left(v\right)\cap V_{n-1}|=2\}$$

and

(2) $$B=\{v\in N_{n}\left(u_{0}\right)\cap D_{3}:|N\left(v\right)\cap V_{n-1}|=1\}.$$

Analogous to the proof of Case 2 in Lemma 4, we have:

**Lemma** **5.** *Let n≥7. Suppose that md(HDN1(n))=3 and $W=\{w_{1},w_{2},w_{3}\}$ is a resolving set of HDN1(n), then we have $w_{i}\notin A$.*

**Lemma** **6.** *Let n≥7. Suppose that md(HDN1(n))=3, $W=\{w_{1},w_{2},w_{3}\}$ is a resolving set of HDN1(n) and $C=N_{n}\left(u_{0}\right)-\left(A\cup B\right)$, then W∩C=∅.*

**Proof.**  Assume $v\in D_{3}$ and $N_{1}\left(v\right)=\left\{u_{1},u_{2},u_{3}\right\}$. Let $h:V\left(G\right)\to Z^{2}$ be a function. Let h(v)=(α,β) and $h\left(u_{i}\right)=\left(\alpha_{i},\beta_{i}\right),$ for 1≤i≤3. We list seven properties Qj, 1≤j≤7 as follows (If Qj holds, we say that (h,v) satisfies Qj):

**(Q1)**  If $u_{i}\in N\left(v\right),$ for 1≤i≤3, then $\alpha \geq \alpha_{i}$ and $\beta \geq \beta_{i}$.
**(Q2)**  $\alpha_{i}=\alpha +1$, for i=1,2,3.
**(Q3)**  $\beta_{i}=\beta +1$, for i=1,2,3.
**(Q4)**  There are at most two vertices $s_{1},s_{2}\in N_{n}\left(u_{0}\right)\cap D_{3}$ such that neither $\left(h,s_{1}\right)$ nor $\left(h,s_{2}\right)$ satisfy Q1.
**(Q5)**  If $v\in N_{n}\left(u_{0}\right)\cap D_{3}$ does not satisfy Q1, then *v* satisfies either Q2 or Q3.
**(Q6)**  For any $v\in N_{n}\left(u_{0}\right)\cap D_{3}$, it is impossible for *v* to satisfy both Q2 and Q3.
**(Q7)**  Given any $v\in N_{n-1}^{-}\left(u_{0}\right)\cap D_{3}$, then *v* satisfies Q1.

 ☐

See Figure 4a.

We have Claim 2 as follows:

**Claim** **2.** *Let $T=W\cap V(G^{\prime })\cap C$. For any subgraph $G^{\prime }$ of HDN1(n) with T≠∅, we assume without loss of generality that $w_{3}\in T$. If $d_{G^{\prime }}\left(u,w_{3}\right)=d_{G}\left(u,w_{3}\right)$ for any $u\in V(G^{\prime })$, then there exists a two-vector coloring scheme on $G^{\prime }$ with respect to vertex $w_{3}$ such that $\left(h,w_{3}\right)$ satisfies Q4–Q7.*

**Proof** **of** **Claim** **2:** Assume $\varphi \left(u,w_{3}\right)=\xi \left(u|W\right)-\xi \left(w_{3}|W\right)=\left(\varphi_{1},\varphi_{2},\varphi_{3}\right)$ for any vertex *u* in HDN1(n). Define a function $h:V\left(G\right)\to Z^{2}$ with $h\left(u\right)=\left(\varphi_{1},\varphi_{2}\right)$ for any vertex *u* in HDN1(n). By the proof of Corollary 1, we know that h(u) satisfies properties (i)–(iii) with k=3 in Definition 1. Therefore the function *h* is a two-vector coloring scheme on $G^{\prime }$. Now we will show that $\left(h,w_{3}\right)$ satisfies Q4–Q7 in the following.

It can be seen that a vertex $v\in D_{3}$ such that (h,v) does not satisfy Q1 if and only if v∈W. Since $w_{3}\in W\cap C$ and |W|=3, then there are at most two vertices $s_{1},s_{2}\in N_{n}\left(u_{0}\right)\cap D_{3}$ such that neither $\left(h,s_{1}\right)$ nor $\left(h,s_{2}\right)$ satisfy Q1. Therefore, we have $\left(h,w_{3}\right)$ satisfies Q4.

Assume $v\in D_{3}$ and *v* does not satisfy Q1, i.e., $v\in \{w_{1},w_{2},w_{3}\}$. It is clear that $v\in \{w_{1},w_{2}\}$. Note that $h\left(u\right)=(d\left(u,w_{1}\right)-d\left(w_{3},w_{1}\right),d\left(u,w_{2}\right)-d\left(w_{3},w_{2}\right))$ for any vertex *u* in HDN1(n). If $v=w_{1}$, then $h\left(v\right)=(-d\left(w_{3},w_{1}\right),d\left(w_{1},w_{2}\right)-d\left(w_{3},w_{2}\right))$ and $h\left(u_{i}\right)=\left(1-d\left(w_{3},w_{1}\right),d\left(u_{i},w_{2}\right)-d\left(w_{3},w_{2}\right)\right)$ for 1≤i≤3. Therefore we have $\alpha_{i}=\alpha +1$, for i=1,2,3, i.e., $\left(h,w_{3}\right)$ satisfies Q2. If $v=w_{2}$, then $h\left(v\right)=(d\left(w_{2},w_{1}\right)-d\left(w_{3},w_{1}\right),-d\left(w_{3},w_{2}\right))$ and $h\left(u_{i}\right)=\left(d\left(u_{i},w_{1}\right)-d\left(w_{3},w_{1}\right),1-d\left(w_{3},w_{2}\right)\right)$ for 1≤i≤3. Therefore we have $\beta_{i}=\beta +1$, i=1,2,3, i.e., $\left(h,w_{3}\right)$ satisfies Q3. Consequently, we have $\left(h,w_{3}\right)$ satisfies Q5.

Suppose $\left(h,w_{3}\right)$ does not satisfy Q6. Then there exists $v\in N_{n}\left(u_{0}\right)\cap D_{3}$ for which (h,v) satisfies Q2 and Q3. Since $\alpha_{i}=\alpha +1$ for i=1,2,3, we have $v=w_{1}$. By $\beta_{i}=\beta +1$ for i=1,2,3, we have $v=w_{2}$. Therefore, we have $w_{1}=w_{2}$, a contradiction.

By Lemma 4, we have that $\left(h,w_{3}\right)$ satisfies Q7 and the proof of Claim 2 is complete.

**Figure 4:**
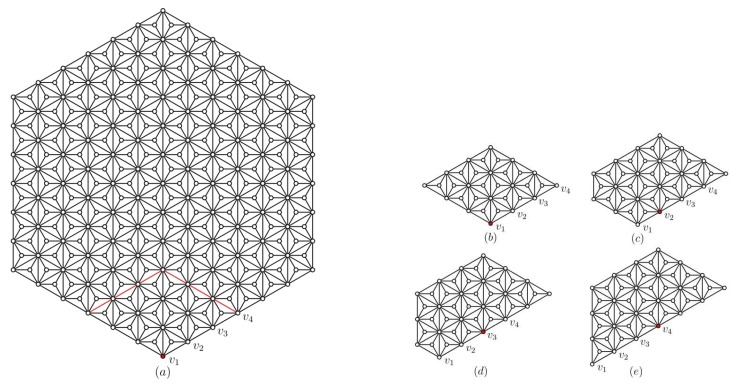
Left: HDN1(7) (**a**); Right: Some vertices of HDN1(7) (**b**–**e**).

If n≥7, as shown in Figure 4b–e, it can be seen that there are only four distinct cases of $N_{3}^{-}\left(w\right)$ for w∈C in HDN1(n). By means of computer search, we have that there exists no two-vector coloring scheme *h* on subgraph $N_{3}^{-}\left(w\right)$ in HDN1(n), for which (h,w) satisfies Q4–Q7. By Claim 2, we have Lemma 6 holds.  ☐

**Lemma** **7.** *Let n≥7. Suppose that md(HDN1(n))=3 and $W=\{w_{1},w_{2},w_{3}\}$ is an RS of HDN1(n), then we have $w_{i}\notin B$.*

**Figure 5:**
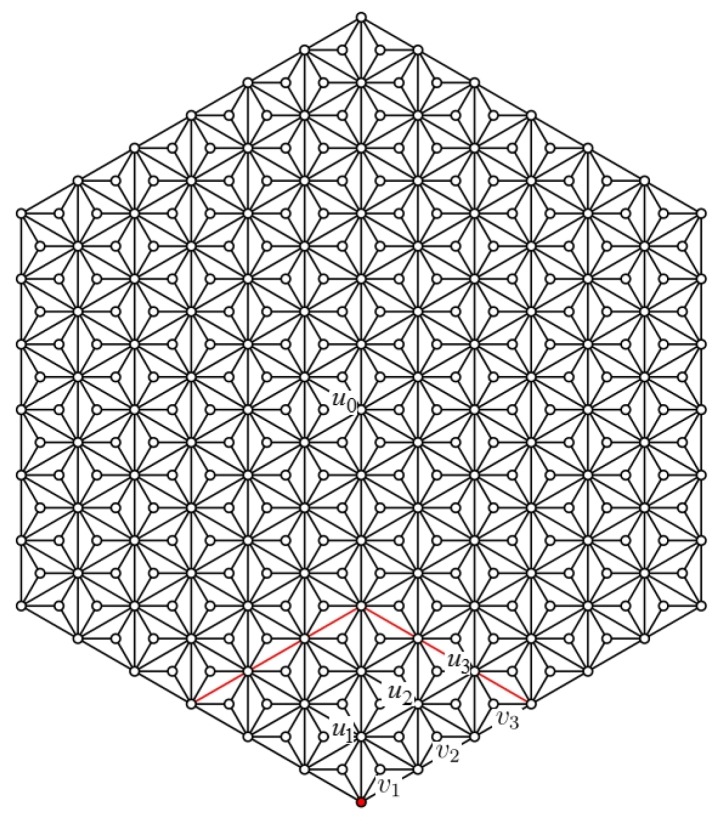
HDN1(n) for $w_{i}\notin B$.

**Proof.**  The proof is by induction on *n*. The statement holds for HDN1(7), which can be confirmed by computer search. Let n≥8 and let the statement hold for all HDN1(m) with m≤n−1. Suppose the statement does not hold for HDN1(n). By Lemmas 4–6, all vertices of any basis *W* are in *B*. On the other hand, for any ordered set $W=\{w_{1},w_{2},w_{3}\}$ and any vertex *v* in HDN1(n), let ξ(v|W) be a metric representation of *v* with respect to *W*. For any $v_{i}\in B$, since $|N\left(v_{i}\right)\cap N\left(V_{n-1}\right)|=1$, we assume $N\left(v_{i}\right)\cap N\left(V_{n-1}\right)=u_{i}$. (See Figure 5).

Then for $w_{i}\in W\cap B$, without loss of generality we have $d\left(v,w\right)=d\left(v,v_{i}\right)=d\left(v,u_{i}\right)+1$ and $u_{i}$ is in HDN1(n−1) for i=1,2,3. Therefore we can obtain a vertex $u_{i}$ with respect to each vertex $w_{i}$ for 1≤i≤3. If $u_{1},u_{2},u_{3}$ are distinct, for any $u\in N_{n-1}^{-}\left(u_{0}\right)$, we have $\left(d\left(u,w_{1}\right),d\left(u,w_{2}\right),d\left(u,w_{3}\right)\right)\neq \left(d\left(t,w_{1}\right),d\left(t,w_{2}\right),d\left(t,w_{3}\right)\right)$ for t∈V(HDN1(n−1)) with t≠u. Consequently, *S* is also a basis of HDN1(n − 1), which yields a contradiction. If $u_{1},u_{2},u_{3}$ are not distinct, then md(HDN1(n − 1))≤2, which yields a contradiction with md(HDN1(n−1))=3.  ☐

Now, we have

**Theorem** **3.** *If n≥2, then md(HDN1(n))=4.*

**Proof.**  For the case n≤6, the results can be verified by solving the instances constructed from an integer linear program introduced in Reference [2]. Suppose the statement does not hold for n≥7, and we assume that $W=\{w_{1},w_{2},w_{3}\}$ is a base of HDN1(n). By Lemmas 4–7, we have W⋂V(HDN1(n))=∅, a contradiction. This completes the proof.  ☐

## 4. Conclusions

In this paper, we provide a proof to show that md(HDN1(n))=4 for n≥2, this indicates that in this type of hex-derived sensor network, the least number of nodes needed to locate any other node is four. This solves an interesting open problem proposed in References [18,22].

## Figures and Tables

**Table 1:** The symbols used in this paper.

Symbol	Definition
$R^{k}$	*k*-dimensional Euclidean space
Z	the set of all integers
$Z^{k}$	$Z^{k}=\left\{\left(x_{1},x_{2},\cdots ,x_{k}\right)\in R^{k}|x_{i}\in Z,1\leq i\leq k\right\}$
$d(v_{1},v_{2})$	the edge number of the shortest path from $v_{1}$ to $v_{2}$
$N_{t}\left(v\right)$	the *t*-neighbourhood of *v*, i.e., $N_{t}\left(v\right)=\left\{s\in V\left(G\right)|d\left(v,s\right)=t\right\}$
N(v)	the *open neighborhood* of *v*, i.e., $N\left(v\right)=N_{1}\left(v\right)$
N[v]	the *closed neighborhood* of *v*, i.e., N[v]=N(v)∪{v}
deg(v)	the degree of *v*
$\xi \left(t|S\right)=(d\left(t,s_{1}\right),d\left(t,s_{2}\right),\cdots ,d\left(t,s_{k}\right))$	the *metric representation of v with respect to S*, where $S=\left\{s_{1},s_{2},\cdots ,s_{k}\right\}\subseteq V\left(G\right)$ is an ordered set
RS	resolving set
md(G)	the metric dimension of *G*
